# Early analgesic response to interventional pain procedures in hospitalized patients with cancer-related pain: clinical predictors and exploratory survival analysis

**DOI:** 10.1007/s00520-026-11004-2

**Published:** 2026-09-08

**Authors:** Joon Hee Lee, Jiwon Yoon, Byunghun Min, Minhye Chang, Francis Sahngun Nahm, Pyung Bok Lee, Eun Joo Choi

**Affiliations:** 1 Department of Anesthesiology and Pain Medicine, Seoul National University Bundang Hospital, 82, Gumi-Ro 173 Beon-Gil, Bundang-Gu, Seongnam, 13620 Korea; 2 Department of Anesthesiology and Pain Medicine, Seoul National University College of Medicine, Seoul, Korea

**Keywords:** Analgesics, opioid, Cancer-related pain, Pain measurement, Pain management, Survival rates

## Abstract

**Purpose:**

Interventional pain procedures (IPPs) are increasingly considered earlier for cancer-related pain, yet the clinical predictors and prognostic relevance of early analgesic response to IPP remain unclear. We aimed to identify predictors of early response and evaluate its implication for 1-year survival outcomes.

**Methods:**

This retrospective cohort study included 349 hospitalized cancer patients undergoing their first inpatient IPP. Early analgesic response was defined by changes in daily mean NRS scores and morphine equivalent daily dose (MEDD) over a 48-h peri-procedural period. Based on these criteria, patients were categorized into overall responders and non-responders. Independent predictors of this early response were identified using multivariable logistic regression, and 1-year survival outcomes were evaluated using Kaplan–Meier analysis with the log-rank test and multivariable Cox proportional hazards regression.

**Results:**

Among 349 patients, overall responders (25.5%) were independently associated with higher baseline NRS (odds ratio [OR], 2.13; 95% confidence interval [CI], 1.63–2.83; *P* < 0.001) and lower baseline MEDD (OR, 0.95 per 10-mg increase; 95% CI, 0.92–0.98; *P* < 0.001). In multivariable Cox regression, early analgesic response was not independently associated with 1-year mortality (adjusted hazard ratio for non-responders vs. overall responders, 1.26; 95% CI, 0.90–1.77; *P* = 0.176), although univariable analysis indicated a significant survival advantage (log-rank test, *P* = 0.037).

**Conclusion:**

Higher pain intensity and lower opioid use predict a favorable early analgesic response to IPP, suggesting an optimal therapeutic window for cancer-related pain intervention. Early analgesic response to IPP may serve as an exploratory prognostic indicator in patients with cancer-related pain.

**Supplementary Information:**

The online version contains supplementary material available at https://doi.org/10.1007/s00520-026-11004-2.

## Introduction

Cancer-related pain often involves multiple overlapping pain mechanisms, including nociceptive and neuropathic components, which contributes to its complexity and challenges in management. The overall prevalence of pain among cancer patients encompasses 44.5%, and that of moderate-to-severe pain in 30.6%; notably, this systemic burden is more pronounced in advanced or metastatic disease [1]. However, cancer-related pain management remains suboptimal in clinical settings, frequently resulting in inadequate symptom control and reduced quality of life [2].

In 1986, the WHO introduced the analgesic ladder as a stepwise pharmacological approach to cancer-related pain management. Traditionally, interventional pain procedures (IPPs), including nerve blocks and neurolytic interventions, were regarded as the final step, and were used only when pharmacological therapies failed [3]. However, accumulating evidence suggests that earlier application of IPPs may lead to superior analgesic outcomes compared with delayed application [4,5], driving a paradigm shift from traditional salvage therapy toward a multimodal approach integrated throughout the continuum of cancer-related pain management [6,7].

Early analgesic response, typically assessed within 24–72 h, is a standard outcome measure in acute pain contexts, such as postoperative or emergency care [8]. In contrast, cancer-related pain is generally managed within a chronic pain framework with greater emphasis on longer-term outcomes, including sustained pain reduction, opioid sparing effects, quality of life, and functional status [7]. Nevertheless, in patients with advanced cancer, early pain reduction following IPPs may provide more than temporary symptomatic relief; it may alleviate physical discomfort and emotional distress [9,10], potentially supporting the continuation of oncological therapies without premature interruption. In this population, evaluating the early post-procedural window is particularly important. This early assessment captures the direct effect of the intervention before outcomes are confounded by rapid adjustments in systemic analgesic regimens or by clinical deterioration leading to loss to follow-up, both of which are frequently encountered in hospitalized patients with advanced malignancies. Given these inherent challenges in long-term palliative care, focusing on this early timeframe provides a practical and clinically meaningful way to evaluate the immediate impact of interventions [11].

Hence, early analgesic response was selected as a pragmatic and clinically relevant outcome measure in this study. Our primary objective was to identify the baseline clinical predictors associated with a favorable early response to inpatient IPP. Furthermore, reflecting the potential link between systemic pain burden and clinical courses of cancer patients [12], we sought to examine, in an exploratory manner, whether achieving this early post-procedural milestone has prognostic significance regarding 1-year survival outcomes. By addressing both clinical predictors and exploratory prognostic implications, our findings offer insights into the broader therapeutic role of IPPs and support their integration into the earlier phases of cancer-related pain management.

## Methods

### Patients

This retrospective cohort study was conducted at the multidisciplinary pain center of Seoul National University Bundang Hospital (SNUBH) and was approved by the Institutional Review Board of SNUBH (B-2208–775-104). The medical records of hospitalized patients with cancer-related pain who were referred for pain management between January 2022 and December 2023 were reviewed. The requirement for informed consent was waived due to the retrospective nature of the study. Patients were included if they met all the following criteria: (1) hospitalization with cancer diagnosis, (2) an index pain source clearly attributable to cancer and distinguishable from non-cancer etiologies, (3) referral to a multidisciplinary pain center for inpatient cancer-related pain management, and (4) no surgical procedure within 14 days prior to referral. To ensure data consistency, this study strictly included inpatient IPPs subsequent to referral during index hospitalization; any outpatient procedures were excluded.

Patients were excluded if (1) no IPP was performed or (2) the procedure was not the first inpatient IPP during the study period, ensuring that only the initial analgesic response per patient was analyzed.

### Outcome measurement

Detailed clinical information was retrospectively collected, including age, sex, height, weight, cancer type and duration, presence of metastasis or bone metastasis (Y/N), cause of hospitalization, Eastern Cooperative Oncology Group Performance Status (ECOG-PS), most clinically significant pain location, and type of IPP performed.

The primary outcome was early analgesic response to IPP, which was evaluated by changes in the mean NRS score (0–10, recorded using a 24-h recall period) and morphine equivalent daily dose (MEDD, mg) measured one day before and after the procedure. The MEDD was calculated using the 2022 CDC Clinical Practice Guidelines for Prescribing Opioids for Pain [13].

Additional outcomes included the occurrence of complications, length of hospital stay, discharge disposition, and 1-year survival status. The reasons for exclusion were recorded. For those excluded due to the absence of IPP, the specific reasons (e.g., patient refusal) were documented.

### Classification of early analgesic response

Early analgesic responses to IPP were classified into four categories: complete response (CR), partial response (PR), pain progression (PP), and indeterminate response (IR). The classification was determined using changes in daily mean NRS scores and MEDD, measured one day before and after IPP, as summarized in Table 1. CR was defined as the complete elimination of pain (post-IPP NRS score = 0) without any concurrent increase in opioid requirement. PR indicated a clinically meaningful improvement, defined as either an absolute reduction in NRS of ≥ 2 points without an increase in MEDD, or a relative decrease in MEDD of ≥ 25% without an increase in NRS. PP referred to a worsening of pain, represented by either an absolute increase in NRS of ≥ 2 points without a decrease in MEDD, or a relative increase in MEDD of ≥ 25% without a decrease in NRS. Patients who did not meet any of these thresholds were classified as having an IR [14].

**Table 1:** Classification of early analgesic response based on changes in NRS and MEDD

Response Category	Absolute change in NRS	Relative change in MEDD
Complete response	Post-IPP NRS = 0 and no increase in MEDD	
Partial response	NRS decreased by ≥ 2 points and no increase in MEDD	MEDD decreased by ≥ 25% and no increase in NRS
Pain progression	NRS increased by ≥ 2 points and no decrease in MEDD	MEDD increased by ≥ 25% and no decrease in NRS
Indeterminate response	Did not meet the criteria above	

The classification was determined using changes in daily mean NRS scores and MEDD, measured one day before and after IPP

*MEDD* morphine equivalent daily dose, *IPP* interventional pain procedure

### Statistical analysis

#### Group comparisons and clinical predictors

The patients were further categorized into overall responders (CR and PR) and non-responders (IR and PP). This dichotomization was used for descriptive purposes and applied consistently to regression and survival analyses to evaluate the clinical predictors and prognostic relevance.

Data are summarized as mean ± SD, median [interquartile range], or number (%), depending on the distribution. Associations between categorical variables were evaluated using the chi-square test or Fisher’s exact test. For continuous variables, Student’s t-test or Mann–Whitney U test was used for group comparisons, while the Wilcoxon signed-rank test was employed to assess within-group changes from baseline.

Multivariable logistic regression analysis was performed using the response status (overall responders vs. non-responders) as a binary outcome variable. Candidate variables were selected based on univariable analyses, with a threshold of *P* < 0.2. Univariable logistic regression analyses were performed for age, sex, height, weight, cancer type, cancer duration, presence of metastasis or bone metastasis, ECOG-PS score, cause of hospitalization, pain location, IPP type, baseline NRS, and baseline MEDD. For categorical variables with more than two levels, the most frequently observed category was designated as the reference group.

#### Survival analyses

Kaplan–Meier survival analyses were performed to estimate the median survival duration and 1-year survival rates for overall responders and non-responders. Survival time was defined as the time from the date of referral to the date of death or administrative censoring at 1 year. For patients lost to follow-up before 1 year, censoring was applied on the date of the last known contact. However, to mitigate the informative censoring bias, patients who transitioned to hospice care were considered to have died by the date of transition. This assumption was based on the clinical course of patients with terminal cancer in the Republic of Korea, for whom hospice transition typically indicates impending death [15]. The difference in survival curves was assessed using the log-rank test. To evaluate the association between early analgesic response and 1-year survival while controlling for potential confounding factors, a subsequent multivariable Cox proportional hazards regression analysis was performed, adjusting for clinically relevant baseline covariates (age, sex, presence of metastasis, baseline NRS, baseline MEDD, and ECOG-PS).

Additionally, to assess the robustness of the main survival model, sensitivity analyses were performed under three alternative assumptions to handle follow-up loss [16].

- Scenario A (standard model): All cases lost to follow-up were censored at the date of the last known contact.
- Scenario B (best-case model): All cases lost to follow-up were assumed to have survived until the end of the 1-year follow-up period.
- Scenario C (worst-case model): All cases lost to follow-up were treated as death events on the date of the last known contact.

In each scenario, Kaplan–Meier survival curves were generated to compare the survival between overall responders and non-responders under the given assumptions. Differences in survival distribution between groups (overall responders vs. non-responders) was assessed using the log-rank test.

Statistical power calculations were not performed a priori as the sample size was determined based on available data. Statistical analyses were performed using the RStudio (v4.3.1; posit PBC, Boston, MA, USA). All statistical tests were two-sided, and statistical significance was set at *P* < 0.05.

## Results

### Study flow and patient inclusion

**Fig. 1:**
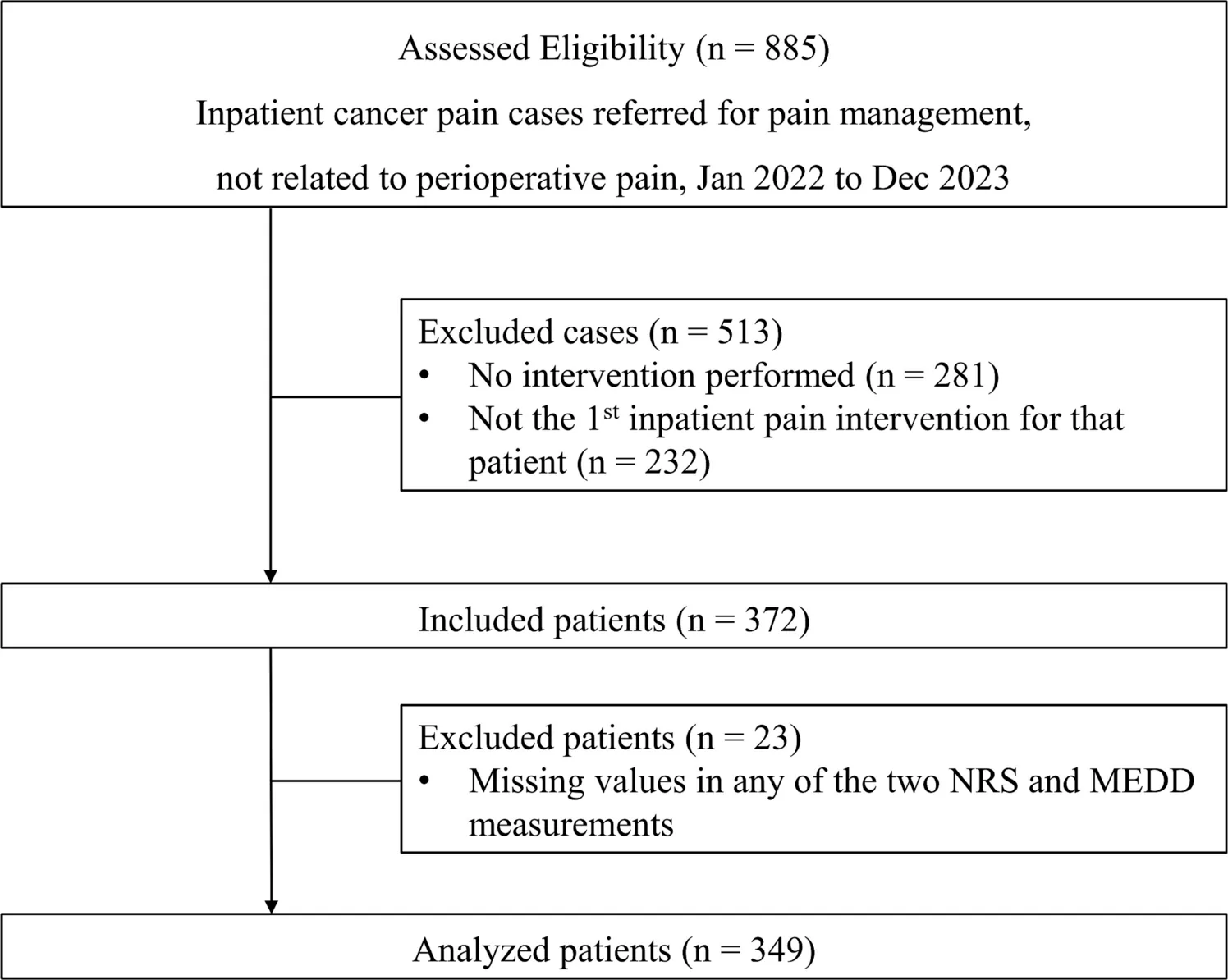
Flow diagram of patients included in this study

Among the 885 inpatient cancer-related pain referral cases, 513 were excluded because no IPP was performed (*n* = 281) or the intervention was not the patient’s first IPP within the study period (*n* = 232). The most common reasons for not undergoing IPP (*n* = 281) were the decision to prioritize pharmacological treatment optimization (36.7%) and patient refusal (11.4%). The remaining 232 cases involved subsequent procedures in 127 patients, and were excluded. Finally, 372 unique patients were identified, of whom 23 were excluded due to missing values in at least one of the required measurements (NRS or MEDD) at baseline or one day post-procedure, and 349 patients were included in the final analysis (Fig. 1).

### Distribution of clinical features, interventions, and outcomes by analgesic response

Among the 349 patients included in the analysis, 25.5% (*n* = 89) were classified as overall responders (CR: 2, PR: 87) and 74.5% (*n* = 260) as non-responders (IR: 221, PP: 39) according to the predefined criteria based on changes in NRS and MEDD (Table 1 and 2).

**Table 2:** Baseline demographic and clinical characteristics by early analgesic response status

	Overall responders (*n* = 89)	Non-responders (*n* = 260)
Age, yr	62.8 ± 12.0	62.5 ± 11.9
Male	52 (58.4)	141 (54.2)
Weight, kg	59.8 ± 15.4	57.9 ± 12.0
Height, cm	162.6 ± 7.9	162.4 ± 8.9
Diagnosis		
Hematologic	2 (2.2)	4 (1.5)
Lung	17 (19.1)	73 (28.1)
Prostate	7 (7.9)	17 (6.5)
Breast	2 (2.2)	16 (6.2)
Genitourinary	6 (6.7)	13 (5.0)
Gynaecologic	5 (5.6)	18 (6.9)
Pancreas	21 (23.6)	35 (13.5)
Colorectal	6 (6.7)	24 (9.2)
Gastric	4 (4.5)	19 (7.3)
Sarcoma	4 (4.5)	6 (2.3)
Hepatobiliary	7 (7.9)	16 (6.2)
Other	8 (9.0)	19 (7.3)
Duration of cancer, months	14.5 [4.0–27.5]	17.0 [7.0–33.0]
Metastasis	79 (88.8)	249 (95.8)
Bone metastasis	50 (56.2)	166 (63.8)
Cause of hospitalization		
Chemotherapy	37 (41.6)	89 (34.2)
Radiotherapy	9 (10.1)	28 (10.8)
Chemo-radiotherapy	4 (4.5)	5 (1.9)
Supportive care	32 (36.0)	107 (41.2)
Work up	6 (6.7)	25 (9.6)
Other	1 (1.1)	6 (2.3)
ECOG-PS		
0	2 (2.2)	0 (0.0)
1	29 (32.6)	85 (32.7)
2	17 (19.1)	77 (29.6)
3	10 (11.2)	25 (9.6)
4	2 (2.2)	2 (0.8)
Missing	29 (32.6)	71 (27.3)
Pain location		
Neck ± Rp	4 (4.5)	8 (3.1)
Upper/Mid back	11 (12.4)	33 (12.7)
Lower back/Pelvis ± Rp	22 (24.7)	88 (33.8)
Trunk	11 (12.4)	31 (11.9)
Abdomen	21 (23.6)	47 (18.1)
Extremity	13 (14.6)	41 (15.8)
Other	7 (7.9)	12 (4.6)
Type of IPP		
ESI	39 (43.8)	155 (59.6)
CPN/SPN	11 (12.4)	15 (5.8)
MBB/FJI	7 (7.9)	16 (6.2)
BPB/Shoulder	9 (10.1)	19 (7.3)
ESPB	11 (12.4)	25 (9.6)
ICNB	2 (2.2)	10 (3.8)
Other	10 (11.2)	20 (7.7)

Data are presented as mean ± SD, median [interquartile range], or number (%)

*ECOG-PS* Eastern Cooperative Oncology Group Performance Status, *Rp* radiating pain, *IPP* interventional pain procedure, *ESI* epidural steroid injection, *CPN* celiac plexus neurolysis, *SPN* splanchnic neurolysis, *MBB* medial branch block, *FJI* facet joint injection, *BPB* brachial plexus block, *ESPB* erector spinae plane block, *ICNB* intercostal nerve block

The baseline demographic and clinical characteristics according to response status are summarized in Table 2. Of overall responders and non-responders, metastatic disease was present in 88.8% and 95.8%, respectively. Poor performance status (ECOG-PS of ≥ 2) was noted in 32.6% of overall responders compared with 40.0% of non-responders, although several patients had missing data. The most common pain location in both groups was lower back/pelvis, with or without radiating pain (24.7% vs. 33.8%), and epidural steroid injection (ESI) was the most frequently performed procedure (43.8% vs. 59.6%).

Table 3 summarizes the pain intensity, opioid use, and clinical outcomes. Both groups showed a significant reduction in NRS scores from baseline (both *P* < 0.001); however, the magnitude of reduction was significantly greater in overall responders than in non-responders (median ΔNRS: − 2.0 vs. 0.0; *P* < 0.001). Regarding opioid dose, MEDD decreased significantly from baseline only in overall responders (*P* < 0.001), resulting in a significant between-group difference in the magnitude of dose reduction (median ΔMEDD: − 15.0 vs. 0.0 mg; *P* < 0.001). The overall incidence of procedural complications across all IPPs was 0.3% (1/349), consisting of a single case of pneumothorax requiring intervention. The corresponding data stratified by the four response categories (CR, PR, IR, and PP) are provided in Supplementary Tables 1–2.

**Table 3:** Pain intensity, opioid use, and clinical outcomes by early analgesic response status

	Overall responders (*n* = 89)	Non-responders (*n* = 260)	*P* value
Pain intensity, NRS			
Baseline	4.0 [4.0–5.0]	4.0 [3.0–4.0]	-
1 day post-procedure	3.0 [2.0–3.0]	3.0 [3.0–4.0]	-
Δ NRS	− 2.0 [− 2.0– − 1.0]^†^	0.0 [− 1.0–0.0]^†^	< 0.001^*^
Opioid use, MEDD			
Baseline	60.0 [26.3–135.0]	90.0 [35.8–165.0]	-
1 day post-procedure	43.8 [15.0–118.8]	104.4 [48.8–180.0]	-
Δ MEDD	− 15.0 [− 30.0–0.0]^†^	0.0 [0.0–24.3]^††^	< 0.001^*^
Complications	0 (0.0)	1 (0.4)	1.000
Hospital day	9.0 [6.0–19.0]	12.0 [7.0–20.0]	0.119
Discharge			0.579
Home	68 (76.4)	184 (70.8)	
Transfer	13 (14.6)	49 (18.8)	
Death	8 (9.0)	27 (10.4)	

Data are presented as median [interquartile range], or number (%). ^*^P value < 0.05

We did not report *P* values for baseline and 1-day post-procedure NRS and MEDD as they were not the primary focus of this table

Within-group changes from baseline to post procedure were analyzed using the Wilcoxon signed-rank test

Δ NRS, ^†^*P*<0.001 for both groups; Δ MEDD, ^†^*P*<0.001 for overall responders and ^††^*P*=1.0 for non-responders

*MEDD* morphine equivalent daily dose

### Predictors of early analgesic response

Univariable and multivariable logistic regression results for factors associated with early analgesic response are presented in Table 4. Variables with *P* < 0.2 in univariable analyses were included as candidate predictors, including duration of cancer, presence of metastasis, type of intervention, baseline NRS, and baseline MEDD. The ECOG-PS yielded a *P* value of 0.067, which met the predefined threshold for inclusion. However, as a substantial proportion of values were missing, the variable was ultimately excluded from the final model to reduce the risk of bias in the adjusted estimates.

**Table 4:** Univariable and multivariable logistic regression analysis for predictors of early analgesic response following interventional pain procedures

	Univariable OR (95% CI)	Univariable *P* value	Multivariable OR (95% CI)	Multivariable* P* value
Age	1.00 (0.98–1.02)	0.843	**-**	**-**
Sex	**-**	0.491	**-**	**-**
Weight	1.01 (0.99–1.03)	0.225	**-**	**-**
Height	1.00 (0.97–1.03)	0.892	**-**	**-**
Cancer diagnosis	-	0.348	**-**	**-**
Duration of cancer	0.99 (0.98–1.00)	0.080^†^	1.00 (0.99–1.01)	0.396
Metastasis	**-**	0.024^†^	0.64 (0.23–1.77)	0.383
Bone metastasis	**-**	0.201	**-**	**-**
Cause of hospitalization	**-**	0.530	**-**	**-**
ECOG-PS	**-**	0.067^†^	**-**	**-**
Pain location	**-**	0.614	**-**	**-**
Type of IPP	**-**	0.151^†^	-	0.266
ESI (Reference)			1.00 (-)	-
CPN/SPN			2.75 (1.00–7.51)	0.047^*^
MBB/FJI			2.01 (0.68–5.52)	0.185
BPB/Shoulder			1.63 (0.60–4.16)	0.317
ESPB			2.01 (0.84–4.62)	0.105
ICNB			0.61 (0.08–2.80)	0.563
Other			1.31 (0.51–3.20)	0.558
Baseline NRS	1.73 (1.37–2.17)	< 0.001^†^	2.13 (1.63–2.83)	< 0.001^*^
Baseline MEDD (per 10 mg)	0.98 (0.96–1.00)	0.019^†^	0.95 (0.92–0.98)	< 0.001^*^

^†^P value < 0.2 for univariable screening (threshold for multivariable model inclusion)

^*^P value < 0.05

ECOG-PS was excluded from the multivariable model because of a high proportion of missing data

The overall *P* value for IPP type indicates the collective significance of the category, while individual *P* values reflect comparisons against the ESI reference group

*OR* odds ratio, *CI* confidence interval, *ECOG-PS* Eastern Cooperative Oncology Group Performance Status, *IPP* interventional pain procedure, *ESI* epidural steroid injection, *CPN* celiac plexus neurolysis, *SPN* splanchnic neurolysis, *MBB* medial branch block, *FJI* facet joint injection, *BPB* brachial plexus block, *ESPB* erector spinae plane block, *ICNB* intercostal nerve block, *MEDD* morphine equivalent daily dose

Early analgesic response was independently associated with higher baseline NRS (odds ratio [OR], 2.13; 95% confidence interval [CI], 1.63–2.83; *P* < 0.001), and lower baseline MEDD (OR, 0.95 per 10-mg increase; 95% CI, 0.92–0.98; *P* < 0.001) (Table 4). Compared with ESI, celiac plexus neurolysis (CPN) or splanchnic neurolysis (SPN) was independently associated with improved analgesic response (OR, 2.75; 95% CI, 1.00–7.51; *P* = 0.047); however, the overall intervention variable was not significantly associated with analgesic response.

### Main survival model and handling of follow-up loss

The main survival model classified patients with confirmed hospice transitions as dead on the date of transition, as described in the Methods section. Among the 349 patients, 186 (53.3%) lacked a complete 1-year follow-up, of whom 133 were confirmed to have entered hospice care. The remaining 53 patients were censored at the date of last known contact.

### Survival outcomes according to early analgesic response

**Fig. 2:**
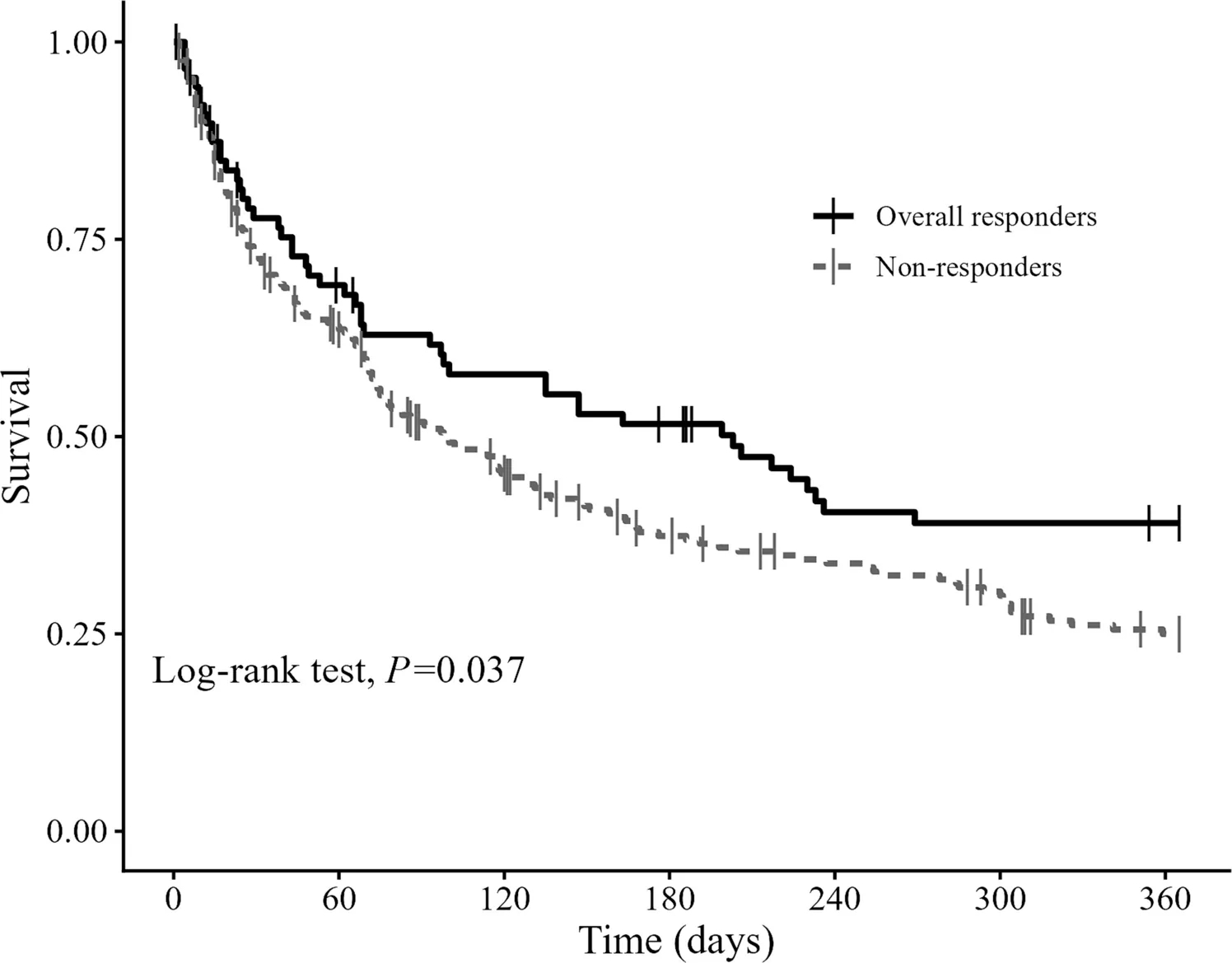
Kaplan–Meier survival curves comparing overall responders and non-responders following cancer-related pain intervention. Patients were classified into two groups based on early analgesic response: overall responders (complete response and partial response) and non-responders (indeterminate response and pain progression). Patients lost to follow-up were censored at the date of the last-known contact. To address informative censoring bias, those with confirmed hospice transition were treated as death events on the date of transition. Overall responders showed significantly better survival throughout the 1-year follow-up period compared with non-responders (log-rank test, *P* = 0.037)

The crude 1-year survival outcomes showed a median survival of 203 days (95% CI, 98 days–not reached) for overall responders and 98 days (95% CI, 75–133 days) for non-responders, with estimated 1-year survival rates of 39.0% and 25.0%, respectively (log-rank test, *P* = 0.037; Fig. 2). However, after adjusting for baseline relevant clinical characteristics specified in Methods (among which ECOG-PS was excluded due to missing data), early analgesic response did not remain independently associated with 1-year mortality (adjusted hazard ratio [aHR] for non-responders vs. overall responders, 1.26; 95% CI, 0.90–1.77; *P* = 0.176). Instead, the multivariable Cox model revealed that the presence of metastasis (aHR, 3.06; 95% CI, 1.34–6.98; *P* = 0.008), higher baseline MEDD (aHR, 1.01 per 10-mg increase; 95% CI, 1.01–1.02; *P* < 0.001), and older age (aHR, 1.02; 95% CI, 1.01–1.03; *P* = 0.001) were independent risk factors for 1-year mortality.

### Sensitivity analyses under alternative censoring assumptions

Kaplan–Meier survival curves comparing overall responders and non-responders under each scenario are presented in Supplementary Fig. 1A–C. The differences in survival distributions were not statistically significant in Scenario A (log-rank test, *P* = 0.13) or Scenario B (log-rank test, *P* = 0.21), but were significant in Scenario C (log-rank test, *P* = 0.026).

## Discussion

This study identifies higher baseline pain intensity and lower baseline opioid use as independent predictors of a favorable early analgesic response following inpatient IPP. Although early analgesic response was associated with better 1-year survival in univariable analysis, this association did not remain independently significant after adjustment for baseline covariates.

During the 2-year study period, 885 patients were referred for inpatient cancer-related pain management, reflecting the substantial demand for pain-related care in this population. Among the final cohort of 349 patients, the predominant cancer types—lung (28.1%), pancreatic (17.3%), and colorectal (9.2%)—align with previous reports demonstrating a high pain burden in these malignancies [17–19]. Most patients presented with advanced-stage disease, as evidenced by the high prevalence of metastasis and bone involvement.

Using our predefined criteria for early analgesic response, 25.5% of the patients were classified as overall responders. Although this proportion appears relatively low, this discrepancy is primarily driven by methodological differences in response definitions. For context, a similar retrospective study by Hochberg et al. [20] evaluated a diverse range of IPPs for cancer-related pain and reported a higher response rate of 42.6% using a ≥ 50% pain relief threshold. While their responder classification relied purely on pain score changes, our criteria integrated both pain intensity dynamics and opioid dose adjustments symmetrically, which naturally lowered the response rate. Notably, the non-responder group demonstrated a significant downward shift in the overall distribution of pain scores, which occurred without a significant change in opioid consumption (Table 3). These findings suggest that even for patients who do not meet the strict threshold for an overall response, IPPs may still provide some degree of short-term pain relief or partial clinical benefit. Overall, IPPs can offer meaningful clinical utility with a very low procedural complication risk (0.3%) for hospitalized patients with cancer-related pain.

The most frequently reported pain sites were the lower back/pelvis (with or without radiating pain) and the abdomen, which are commonly associated with skeletal or visceral involvement [21,22]. The most frequently performed procedure was ESI (55.6%). This high utilization directly reflects the high prevalence of bone metastases (61.9%) or documented axial or truncal pain (59.6%) in our cohort, for which epidural injection was considered a reasonable therapeutic option given the underlying pain mechanism. Previously, ESI has been shown to provide significant pain relief and attenuate opioid escalation in patients with spinal metastases [23]. Although the 20.1% (39/194) response rate observed for ESI in this study may appear numerically lower than rates reported in prior studies, this profile primarily reflects our rigorous composite response criteria and showed no marked difference from the cohort’s overall responder rate of 25.5%. In contrast, neurolytic sympathetic block (e.g., CPN or SPN), which specifically target visceral pain [24], demonstrated a higher response rate of 42.3% (11/26), consistent with previous reports of their efficacy in upper abdominal malignancies [25–27].

Higher baseline pain intensity and lower baseline opioid use emerged as independent predictors of a favorable response to IPP. This combination identifies a critical clinical window where intense pain exists prior to the escalation to high-dose opioid therapy. Chronologically, this threshold aligns with the concept of early intervention, where clinical responsiveness is enhanced by targeting shorter pain durations before chronic neuroplastic changes occur [28]. On a pharmacological level, prolonged high-dose opioid exposure may impair the procedural efficacy. This limitation can be explained by cross-tolerance between opioid and local anesthetics [29–32] or the development of opioid-induced hyperalgesia [33], both of which paradoxically blunt the overall responsiveness to IPP. These results indicate that the optimal therapeutic window for IPP efficacy is determined by both the chronological timing relative to the biological progression of pain and the patient’s pharmacological status before the development of significant opioid-induced sensitization. Consequently, our findings reinforce a paradigm shift in viewing IPPs not as a last resort at the final step of the WHO ladder, but as a proactive strategy of early integration throughout the continuum of cancer-related pain management [6,7, 34],

In the univariable analysis, patients achieving an early analgesic response demonstrated significantly improved survival over the 1-year follow-up period compared with non-responders. However, sensitivity analyses using alternative censoring assumptions showed that this survival advantage was not robust across different scenarios (Supplementary Fig. 1). Furthermore, in the subsequent multivariable Cox model, this association did not remain independently significant after accounting for baseline clinical characteristics. Given these discrepancies, any direct prognostic inference from early analgesic response to long-term survival must be interpreted with caution. Nevertheless, from a theoretical standpoint, effective acute pain control may help mitigate psychological distress and preserve short-term physiological resilience [10, 35], potentially supporting performance status during subsequent oncologic therapies. Accordingly, early analgesic response to IPP may be viewed as an exploratory prognostic indicator, a hypothesis that warrants further prospective validation.

This study had several limitations. First, its retrospective design is inherently prone to selection bias and residual confounding. Second, pain assessment was based on mean NRS scores, which may have masked episodes of breakthrough pain [36]. Third, ECOG-PS, an established prognostic factor [37–39], was frequently missing and insufficiently incorporated into the multivariable analyses. Fourth, this study was conducted in an inpatient setting, which may limit the generalizability of our findings to a broader population of outpatients. Lastly, because our primary outcome focused on early analgesic response, long-term analgesic efficacy could not be assessed.

## Conclusion

Higher baseline pain intensity and lower baseline opioid use were independent predictors of a favorable early analgesic response to inpatient IPP. These findings identify an optimal therapeutic window determined by both chronological timing relative to pain progression and pharmacological status before high-dose opioid escalation. Although early analgesic response was associated with improved crude 1-year survival, this association did not remain independent after multivariable adjustment. Early analgesic response to IPP may therefore serve as an exploratory prognostic indicator in patients with cancer-related pain.

## Supplementary Information

Below is the link to the electronic supplementary material.

ESM 1 Supplementary Material 1: Kaplan–Meier survival curves comparing overall responders and non-responders under alternative censoring assumptions. (A) Scenario A (standard model): All cases lost to follow-up were treated as non-informative censoring at the date of last contact (Log-rank test, *P* = 0.13). (B) Scenario B (best-case model): All cases lost to follow-up were assumed to have survived until the end of the 1-year follow-up period (Log-rank test, *P* = 0.21).(C) Scenario C (worst-case model): All cases lost to follow-up were treated as death events occurring on the date of last-known contact (Log-rank test, *P* = 0.026). (PNG 68.5 KB)

High Resolution Image (TIFF 7.72 MB)

ESM 2 Supplementary Material 2 (PNG 64.1 KB)

High Resolution Image (TIFF 7.72 MB)

ESM 3 Supplementary Material 3 (PNG 73.6 KB)

High Resolution Image (TIFF 7.72 MB)

ESM 4 Supplementary Material 4: Comparison of the main survival model with alternative scenarios for the entire cohort. (A) Main model vs. Scenario A: Comparison with the standard model (Log-rank test, *P* < 0.001). (B) Main model vs. Scenario B: Comparison with the best-case model (Log-rank test, *P* < 0.001). (C) Main model vs. Scenario C: Comparison with the worst-case model (Log-rank test, *P* = 0.01). (PNG 111 KB)

High Resolution Image (TIF 93.6 KB)

ESM 5 Supplementary Material 5 (PNG 95.5 KB)

High Resolution Image (TIF 82.5 KB)

ESM 6 Supplementary Material 6 (PNG 100 KB)

High Resolution Image (TIF 84.8 KB)

ESM 7 Supplementary Material 7 (DOCX 20.0 KB)

ESM 8 Supplementary Material 8 (DOCX 16.3 KB)

## Data Availability

Data will be made available upon reasonable request.
