# Supplementary material for: Early analgesic response to interventional pain procedures in hospitalized patients with cancer-related pain: clinical predictors and exploratory survival analysis
Source: Support Care Cancer. 2026 Sep 8;34(10):945. doi: 10.1007/s00520-026-11004-2 (PMC13553683; doi:10.1007/s00520-026-11004-2)
Supplement: Supplementary file 1 [file 520_2026_11004_MOESM7_ESM.docx]

**Supplementary Table 1.** Baseline demographic and clinical characteristics by early analgesic response categories.

|  | **CR**  **(n=2)** | **PR**  **(n=87)** | **IR**  **(n=221)** | **PP**  **(n=39)** |
| --- | --- | --- | --- | --- |
| **Age (yr)** | 72.5 (7.8) | 62.6 (12.1) | 62.7 (12.1) | 61.3 (11.2) |
| **Male** | 1 (50) | 51 (58.6) | 117 (52.9) | 24 (61.5) |
| **Weight (kg)** | 54.6 (8.1) | 59.9 (15.6) | 57.6 (11.6) | 59.6 (14.1) |
| **Height (cm)** | 161.7 (8.8) | 162.6 (8.0) | 162.2 (8.8) | 163.9 (9.0) |
| **Diagnosis** |  |  |  |  |
| Haematologic | 0 (0.0) | 2 (2.3) | 4 (1.8) | 0 (0.0) |
| Lung | 0 (0.0) | 17 (19.5) | 65 (29.4) | 8 (20.5) |
| Prostate | 0 (0.0) | 7 (8.0) | 12 (5.4) | 5 (12.8) |
| Breast | 0 (0.0) | 2 (2.3) | 14 (6.3) | 2 (5.1) |
| Genitourinary | 0 (0.0) | 6 (6.9) | 13 (5.9) | 0 (0.0) |
| Gynaecologic | 0 (0.0) | 5 (5.7) | 15 (6.8) | 3 (7.7) |
| Pancreas | 2 (100.0) | 19 (21.8) | 26 (11.8) | 9 (23.1) |
| Colorectal | 0 (0.0) | 6 (6.9) | 20 (9.0) | 4 (10.3) |
| Gastric | 0 (0.0) | 4 (4.6) | 16 (7.2) | 3 (7.7) |
| Sarcoma | 0 (0.0) | 4 (4.6) | 6 (2.7) | 0 (0.0) |
| Hepatobiliary | 0 (0.0) | 7 (8.0) | 14 (6.3) | 2 (5.1) |
| Other | 0 (0.0) | 8 (9.2) | 16 (7.2) | 3 (7.7) |
| **Duration of cancer (months)** | 0.5 (0.3–0.8) | 15.0 (5.0–28.5) | 19.0 (7.0–34.0) | 13.0 (7.0–18.0) |
| **Metastasis** | 2 (100.0) | 77 (88.5) | 212 (95.9) | 37 (94.9) |
| **Bone metastasis** | 0 (0.0) | 50 (57.5) | 141 (63.8) | 25 (64.1) |
| **Cause of hospitalization** |  |  |  |  |
| Chemotherapy | 2 (100.0) | 35 (40.2) | 79 (35.7) | 10 (25.6) |
| Radiotherapy | 0 (0.0) | 9 (10.3) | 26 (11.8) | 2 (5.1) |
| Chemo-radiotherapy | 0 (0.0) | 4 (4.6) | 5 (2.3) | 0 (0.0) |
| Supportive care | 0 (0.0) | 32 (36.8) | 84 (38.0) | 23 (59.0) |
| Work up | 0 (0.0) | 6 (6.9) | 22 (10.0) | 3 (7.7) |
| Other | 0 (0.0) | 1 (1.1) | 5 (2.3) | 1 (2.6) |
| **ECOG-PS** |  |  |  |  |
| 0 | 0 (0.0) | 2 (2.3) | 0 (0.0) | 0 (0.0) |
| 1 | 1 (50.0) | 28 (32.2) | 77 (34.8) | 8 (20.5) |
| 2 | 0 (0.0) | 17 (19.5) | 64 (29.0) | 13 (33.3) |
| 3 | 0 (0.0) | 10 (11.5) | 22 (10.0) | 3 (7.7) |
| 4 | 0 (0.0) | 2 (2.3) | 2 (0.9) | 0 (0.0) |
| Missing | 1 (50.0) | 28 (32.2) | 56 (25.3) | 15 (38.5) |
| **Pain location** |  |  |  |  |
| Neck ± Rp | 0 (0.0) | 4 (4.6) | 8 (3.6) | 0 (0.0) |
| Upper/Mid back | 0 (0.0) | 11 (12.6) | 29 (13.1) | 4 (10.3) |
| Low back/Pelvis ± Rp | 0 (0.0) | 22 (25.3) | 78 (35.3) | 10 (25.6) |
| Trunk | 0 (0.0) | 11 (12.6) | 26 (11.8) | 5 (12.8) |
| Abdomen | 2 (100.0) | 19 (21.8) | 39 (17.6) | 8 (20.5) |
| Extremity | 0 (0.0) | 13 (14.9) | 34 (15.4) | 7 (17.9) |
| Other | 0 (0.0) | 7 (8.0) | 7 (3.2) | 5 (12.8) |
| **Type of IPP** |  |  |  |  |
| ESI | 0 (0.0) | 39 (44.8) | 128 (57.9) | 27 (69.2) |
| CPN/SPN | 2 (100.0) | 9 (10.3) | 14 (6.3) | 1 (2.6) |
| MBB/FJI | 0 (0.0) | 7 (8.0) | 16 (7.2) | 0 (0.0) |
| BPB/Shoulder | 0 (0.0) | 9 (10.3) | 17 (7.7) | 2 (5.1) |
| ESPB | 0 (0.0) | 11 (12.6) | 21 (9.5) | 4 (10.3) |
| ICNB | 0 (0.0) | 2 (2.3) | 9 (4.1) | 1 (2.6) |
| Other | 0 (0.0) | 10 (11.5) | 16 (7.2) | 4 (10.3) |

Data are presented as mean (SD), median (interquartile range), or number (%).

CR, complete response; PR, partial response; IR, indeterminate response; PP, pain progression; ECOG-PS, Eastern Cooperative Oncology Group Performance Status, Rp, radiating pain; IPP, interventional pain procedure; ESI, epidural steroid injection; CPN, celiac plexus neurolysis; SPN, splanchnic neurolysis; MBB, medial branch block; FJI, facet joint injection; BPB, brachial plexus block; ESPB, erector spinae plane block; ICNB, intercostal nerve block.
