# Supplementary material for: Early analgesic response to interventional pain procedures in hospitalized patients with cancer-related pain: clinical predictors and exploratory survival analysis
Source: Support Care Cancer. 2026 Sep 8;34(10):945. doi: 10.1007/s00520-026-11004-2 (PMC13553683; doi:10.1007/s00520-026-11004-2)
Supplement: Supplementary file 2 [file 520_2026_11004_MOESM8_ESM.docx]

**Supplementary Table 2.** Pain intensity, opioid use, and clinical outcomes according to early analgesic response categories.

|  | **CR**  **(n=2)** | **PR**  **(n=87)** | **IR**  **(n=221)** | **PP**  **(n=39)** |
| --- | --- | --- | --- | --- |
| **Pain intensity (NRS)** |  |  |  |  |
| Baseline | 3.0 (3.0–3.0) | 4.0 (4.0–5.0) | 4.0 (3.0–5.0) | 4.0 (3.0–4.0) |
| 1 day post-procedure | 0.0 (0.0–0.0) | 3.0 (2.0–3.0) | 3.0 (2.0–4.0) | 4.0 (4.0–5.0) |
| Δ NRS | −3.0 (−3.0–−3.0) | −2.0 (-2.0–−1.0) | −1.0 (−1.0–0.0) | 0.0 (0.0–1.0) |
| **Opioid use (MEDD, mg)** |  |  |  |  |
| Baseline | 18.8 (9.4–28.1) | 60.0 (26.9–136.3) | 94.8 (30.7–172.5) | 75.0 (42.8–120.0) |
| 1 day post-procedure | 18.8 (9.4–28.1) | 43.8 (15.0–119.4) | 101.3 (40.1–180.0) | 148.8 (68.1–211.3) |
| Δ MEDD | 0.0 (0.0–0.0) | −15.0 (−30.0–0.0) | 0.0 (0.0–15.0) | 53.8 (29.5–88.8) |
| **Complications** | 0 (0.0) | 0 (0.0) | 1 (0.4) | 0 (0.0) |
| **Hospital day** | 8.0 (7.5–8.5) | 10.0 (6.0–19.0) | 12.0 (7.0–20.0) | 13.0 (10.0–20.0) |
| **Discharge** |  |  |  |  |
| Home | 2 (100.0) | 66 (75.9) | 160 (72.4) | 24 (61.5) |
| Transfer | 0 (0.0) | 13 (14.9) | 41 (18.6) | 8 (20.5) |
| Death | 0 (0.0) | 8 (9.2) | 20 (9.0) | 7 (17.9) |

Data are presented as median (interquartile range), or number (%).

CR, complete response; PR, partial response; IR, indeterminate response; PP, pain progression; MEDD, morphine equivalent daily dose.
